# Proteomic characterisation of prostate cancer intercellular communication reveals cell type-selective signalling and TMSB4X-dependent fibroblast reprogramming

**DOI:** 10.1007/s13402-022-00719-z

**Published:** 2022-09-28

**Authors:** Yunjian Wu, Kimberley C. Clark, Elizabeth V. Nguyen, Birunthi Niranjan, Lisa G. Horvath, Renea A. Taylor, Roger J. Daly

**Affiliations:** 1 Cancer Program, Biomedicine Discovery Institute, Monash University, Clayton, VIC 3800 Australia; 2 Department of Biochemistry and Molecular Biology, Monash University, Clayton, VIC 3800 Australia; 3 Department of Anatomy and Developmental Biology, Monash University, Clayton, VIC 3800 Australia; 4 Garvan Institute of Medical Research, Darlinghurst, NSW 2010 Australia; 5 University of Sydney, Camperdown, NSW 2006 Australia; 6 Chris O’Brien Lifehouse, Camperdown, NSW 2050 Australia; 7 Department of Physiology, Monash University, Clayton, VIC 3800 Australia; 8 Cancer Research Division, Peter MacCallum Cancer Centre, The University of Melbourne, Melbourne, VIC 3800 Australia

**Keywords:** Tumour microenvironment, Cell signalling, Actin cytoskeleton

## Abstract

**Background:**

In prostate cancer, the tumour microenvironment (TME) represents an important regulator of disease progression and response to treatment. In the TME, cancer-associated fibroblasts (CAFs) play a key role in tumour progression, however the mechanisms underpinning fibroblast-cancer cell interactions are incompletely resolved. Here, we address this by applying cell type-specific labelling with amino acid precursors (CTAP) and mass spectrometry (MS)-based (phospho)proteomics to prostate cancer for the first time.

**Methods:**

Reciprocal interactions between PC3 prostate cancer cells co-cultured with WPMY-1 prostatic fibroblasts were characterised using CTAP-MS. Signalling network changes were determined using Metascape and Enrichr and visualised using Cytoscape. Thymosin β4 (TMSB4X) overexpression was achieved via retroviral transduction and assayed by ELISA. Cell motility was determined using Transwell and random cell migration assays and expression of CAF markers by indirect immunofluorescence.

**Results:**

WPMY-1 cells co-cultured with PC3s demonstrated a CAF-like phenotype, characterised by enhanced PDGFRB expression and alterations in signalling pathways regulating epithelial-mesenchymal transition, cytoskeletal organisation and cell polarisation. In contrast, co-cultured PC3 cells exhibited more modest network changes, with alterations in mTORC1 signalling and regulation of the actin cytoskeleton. The expression of the actin binding protein TMSB4X was significantly decreased in co-cultured WPMY-1 fibroblasts, and overexpression of TMSB4X in fibroblasts decreased migration of co-cultured PC3 cells, reduced fibroblast motility, and protected the fibroblasts from being educated to a CAF-like phenotype by prostate cancer cells.

**Conclusions:**

This study highlights the potential of CTAP-MS to characterise intercellular communication within the prostate TME and identify regulators of cellular crosstalk such as TMSB4X.

**Supplementary Information:**

The online version contains supplementary material available at 10.1007/s13402-022-00719-z.

## Background

Prostate cancer (PC) is the second most common malignancy in men worldwide, and is associated with high morbidity and mortality in patients with high-risk disease. There is an urgent need to dissect the pathophysiological mechanisms of PC progression, which will enable the development of new treatment strategies. While the vast majority of research on PC has focused on malignant epithelial cells, it is evident that the tumour microenvironment (TME), comprised of extracellular matrix (ECM) and diverse cell types, plays a major role in cancer development and progression [1, 2]. Stromal fibroblasts, including carcinoma-associated fibroblasts (CAFs), are a predominant population of cells in the TME and there is convincing evidence that they actively promote progression to advanced disease [3–5]. CAFs isolated from human prostatic specimens stimulate cancer cell proliferation, increase neovascularisation within the tumour and enhance epithelial-mesenchymal transition (EMT) to facilitate metastasis [3, 6]. CAFs communicate with each other, the other cells within the TME as well as cancer cells in a reciprocal manner via soluble mediators and paracrine signalling. Further understanding the complex signalling mechanisms underpinning tumour stroma-cancer cell interactions during PC progression will enable the identification of novel biomarkers and/or treatment strategies.

To date, studies dissecting the signalling mechanisms involved in fibroblast-cancer cell interactions have largely involved isolated cultures of cancer cells or human prostatic fibroblasts [7, 8]. Powerful MS-based (phospho)proteomic profiling platforms have then been employed to provide insights into cancer signalling networks in individual cell types [9]. Using this approach, we previously identified a CAF-signalling pathway involving lysyl oxidase-like 2 (LOXL2), which crosslinks collagen, specific fibrillary collagens, and their cell surface receptor DDR2, as an important CAF-mediated signalling axis that contributes to prostate cancer progression and is a potential therapeutic target [9]. While 2D and 3D methods have been developed to simultaneously grow stromal fibroblasts with cancer cells, it has not been possible to define the signalling events intrinsic to the different cell types in co-culture since the methodologies employed could not discriminate between DNA, RNA or protein from the different cell types. Previous attempts used cell sorting to isolate the individual cell populations from co-cultures prior to analysis, but this will perturb intercellular signalling and associated short-lived post-translational modifications such as phosphorylation [10].

To overcome this technical limitation, we employed the cell type-specific labelling with amino acid precursors (CTAP)-mass spectrometry (MS) approach, which allows the proteomes of two different cell types in co-culture to be readily distinguished by MS [10]. This has been used previously to demonstrate that oncogenic KRAS signalling in pancreatic cancer cells not only acts in a cell-autonomous fashion, but also indirectly, via reciprocal interactions with co-cultured pancreatic stellate cells, which in turn increase cancer cell proliferation, reduce apoptosis and alter mitochondrial activity [11, 12]. Thus, CTAP has the capacity to reveal both cell-autonomous and reciprocal, intercellular signalling pathways in the TME, thus unveiling potential therapeutic targeting strategies.

In this study, we established co-cultures of immortalised (WPMY-1) prostatic fibroblasts [13] and PC3 prostate cancer cells as a model system to study intercellular communication between prostate cancer cells and stromal fibroblasts. Having demonstrated that WPMY-1 cells promoted cell proliferation and migration of the PC3 cells, we exploited CTAP to characterise reciprocal communication between the two cell types. These data identified alterations in key signalling networks in the two cell types and also down-regulation of the actin-binding protein Thymosin β4 (TMSB4X) in the WPMY-1 cells as a key regulatory event in intercellular crosstalk, providing important mechanistic and functional insights into the biology of the prostate cancer TME.

## Methods

### Cell culture and patient samples

PC3, WPMY-1 and HEK293T cells were obtained from ATCC (Manassas, VA, USA). PC3 and WPMY-1 cells were cultured in RPMI 1640 medium (School of Biomedical Sciences, Media and Prep Services, Monash University) supplemented with 5% (v/v) fetal bovine serum (FBS, Invitrogen, Waltham, MA, USA). HEK293T cells were cultured in DMEM medium (School of Biomedical Sciences, Media and Prep Services, Monash University) supplemented with 5% (v/v) FBS. All cell lines tested negative for Mycoplasma and were validated via short tandem repeat (STR) profiling by Cell Bank Australia.

### Fluorescence/luciferase transfection

The expression vector for Luciferase/mCherry (pRV100G ofl T2A Luciferase/mCherry) was kindly provided by Prof. Paul Timpson (Garvan Institute, NSW, Australia). Expression vector pGIPZ for GFP was purchased from Thermo Fisher Scientific (Waltham, MA, USA). Lentiviral or retroviral transduction of PC3 and WPMY-1 cells for GFP, mCherry and Luciferase expression was performed as previously described [14] by using Lipofectamine™ 3000 reagent (Thermo Fisher Scientific). Positive cells were selected by Fluorescence-activated cell (FAC) sorting.

### TMSB4X overexpressing WPMY-1 cells

The coding region of the human TMSB4X cDNA (GenBank accession no. NM_021109.3) ± Flag tag was inserted into the pMIG (MSCV-IRES-GFP) retrovirus vector by using NEBuilder HiFi DNA Assembly Master Mix (New England Biolabs, Ipswich, MA, USA) and validated by DNA sequencing. HEK293T cells were transiently transfected with pMIG_TMSB4X ± Flag tag retrovirus and appropriate protein expression confirmed by Western blot. Then, the WPMY-1 cells were transfected with pMIG alone or with pMIG _TMSB4X cDNA ± Flag. Positive cells were selected by FAC sorting for GFP.

### Transfection and cell-type labelling with amino acid precursors

The expression vectors for Proteus mirabilis lysine racemase (M4GGR9) (SFFV-EGFP-T2A-Lyr_M37-KDEL-HA, Lyr^M37−KDEL^) and Mycobacterium tuberculosis (P0A5M4) diaminopimelate decarboxylase (DDC) (SFFV-mCherry-T2A-DDC_Mtub-KDEL-HA, DDC^M.tub−KDEL^) were obtained from Dr Claus Jørgensen (Cancer Research UK Manchester Centre). The PC3 and WPMY-1 cells were infected with lentivirus (ViraPower™ Lentiviral Expression System, Invitrogen) for Lyr^M37−KDEL^ and DDC^M.tub−KDEL^ expression, respectively. PC3-Lyr^M37−KDEL^ cells were grown in the basic SILAC medium (RPMI1640 (deficient for L-lysine and L-arginine) (DMP49, Caisson, UT, USA) supplemented with 10% (v/v) dialysed FBS (Invitrogen) and 0.3 mM L-arginine (A8094, Sigma-Aldrich, MO, USA)) with 2.5 mM heavy D-lysine-3,3,4,4,5,5,6,6-d8 2HCl (C/D/N D-6367). WPMY-1-DDC^M.tub−KDEL^ cells were grown in the basic SILAC medium with 5 mM diaminopimelate (DAP).

### Western blot analysis

Standard Western blots were undertaken using RIPA lysates as previously described [15]. For TMSB4X detection, due to its small size (7 kDa), different conditions for each step of western blot have been optimised. In detail, RIPA buffer, 1 × and 2 × loading buffer (4% (v/v) SDS) were used to lyse the cells, and proteins were separated on 12%, 15% and 20% (v/v) polyacrylamide gels, respectively. Proteins were electrotransfered at 30 V, 60 V, 90 V and 110 V at room temperature or in cold room for 10 min, 20 min and 30 min, respectively. The PVDF membranes were activated by either 100% methanol or 100% ethanol before electrotransfer, and were fixed by methanol or paraformaldehyde or no fixation after electrotransfer.

The final optimised condition was to directly harvest and lyse the cells using 2 × loading buffer (4% (v/v) SDS), and 10 to 20 μg of total cellular extracts were separated on 15% (v/v) SDS polyacrylamide gels. Electrotransfer was performed in a cold room at 90 V using Immuno-blot PVDF membranes (0.2 μm). Prior to electrotransfer, PVDF membranes were treated with 100% methanol for 15 s, transferred to a container of distilled water for 2 min, and then soaked with the transfer buffer for 15 min. After electrotransfer, PVDF membranes were soaked at room temperature for 30 min with either 100% methanol or 1 × PBS containing 0.4% (v/v) paraformaldehyde [16].

### Enzyme-linked immunosorbent assay (ELISA)

TMSB4X was assayed using a human TMSB4X ELISA kit (RayBiotech, Norcross, GA, USA). All samples were processed in duplicate following the manufacturer’s instructions. The detection ranges were between 0.1–1000 ng/ml.

### Preparation of conditioned medium

Cells were seeded in 10 cm culture dishes at 1.6 × 10^6^ cells/dish in complete medium. Upon reaching 80–90% confluence, cells were washed with PBS and the medium replaced with 8 ml serum-free medium. After 48 h, the conditioned medium was collected, centrifuged and stored at -80 ℃ until the analysis was performed.

### Proliferation assay for cells in co-culture

PC3-GFP cells (5000/well) or PC3-GFP + WPMY-1 cells (5000 + 5000/well) were seeded in a 96 well plate and cultured for 3 days. The number of GFP positive cells was counted using ImageJ software.

### MTS proliferation assay for cells in conditioned medium

The effect of the conditioned medium on the proliferation of PC3 cells was performed by MTS assay. Briefly, PC3 cells were seeded in a 96-well plate at 5000 cells per well. At 0 h, 24 h, 48 h and 72 h time points, 20 µl of MTS reagent (CellTiter 96 Aqueous One Solution MTS assay reagent, Promega, Wisconsin, USA) was added to the culture and incubated for 60 min. Absorbance was measured using a CLARIOstar plate reader (BMG Labtech) as a measure of cell number.

### Random cell migration assay

PC3-mCherry cells (1.8 × 10^4^/well), WPMY-1-GFP cells with/without TMSB4X overexpression (1.8 × 10^4^/well) or PC3-mCherry cells + WPMY-1-GFP cells with/without TMSB4X overexpression (1.8 × 10^4^ + 1.8 × 10^4^/well) were seeded in a 12 well plate. After 24 h, the medium was replaced with serum-free medium with 1 μg/ml Mitomycin C (Sigma-Aldrich). Random cell migration was monitored using a Leica DMi8 Live Cell microscope (Wetzlar, Germany). Three fields per well were chosen under a 10 × magnification objective. Time-lapse movies of the PC3-mCherry and WPMY-1-GFP cells in each mono-culture and co-culture condition were recorded over 24 h with an image acquired every 20 min. The movie files were analysed using the MtrackJ plug-in of the ImageJ software.

### Transwell migration assay

Transwell migration assays were performed using a 24-well Transwell plate (8 μm pore size, Millipore, Burlington, MA, USA). 4 × 10^4^ PC3 cells were suspended in 100 μl serum free RPMI 1640 medium and added to the upper chamber. The lower chamber was filled with WPMY-1 conditioned medium or serum-free RPMI 1640 medium (basal medium) as the control. After 24 h of incubation, the cells remaining in the upper chamber were removed by cotton swabs. Cells on the lower surface of the membrane were fixed in 4% (v/v) paraformaldehyde and stained with crystal violet. Cells in five microscopic fields (at 4 × magnification) were counted and photographed. All experiments were performed in triplicate.

### Immunofluorescence staining

Cells were seeded onto coverslips, and the growth media was removed by 3 × PBS washes using a Pasteur pipette. Cells were fixed with 4% (v/v) formaldehyde/PBS for 20 min at room temperature. Fixed cells were permeabilised with 0.1% (v/v) Triton X-100 in PBS for 2 min. Then the coverslips were completely immersed in blocking solution (1% (w/v) BSA) for 25 min. Then, cells were probed with either α-SMA (#19,245, Cell Signalling Technology) or PDGFRB (#3169, Cell Signalling Technology) antibodies in 1% BSA for 1 h and then washed 3 × with PBS and once with 1% (w/v) BSA in PBS. Cells were incubated with Alexa-fluor488-conjugated or Alexa-fluor594-conjugated secondary antibodies (Life Technologies, Carlsbad, CA, USA) in 1% (w/v) BSA for 1 h and then washed with PBS. Nuclei were counterstained with 0.1 μg/ml of 4′, 6-diamindino-2-phenylindole (DAPI, Life Technologies). Coverslips were then mounted onto glass slides using 4 µl of Fluoromount mounting media and visualised via confocal microscopy. Images were acquired using a Nikon C1 inverted confocal microscope using ACT-1 software (Nikon corporations, Tokyo, Japan) and processed by ImageJ software.

### CTAP co-culture

“Heavy” PC3-Lyr^M37−KDEL^ cells were co-cultured with “light” WPMY-1-DDC^M.tub−KDEL^ cells in a 1:1 ratio. Specifically, 1 × 10^6^ PC3-LyrM37-KDEL cells and 1 × 10^6^ WPMY-1-DDCM.tub-KDEL cells were seeded onto a 15 cm dish in co-culture SILAC media (basic SILAC medium + D-lysine + DAP). In parallel, 1 × 10^6^ PC3-LyrM37-KDEL cells and 1 × 10^6^ WPMY-1-DDCM.tub-KDEL cells were separately seeded onto 15 cm dishes as mono-cultures. Cells were allowed to grow for 5 days.

### Mass spectrometry

#### Sample preparation

At the end of the culture period, cells were washed 2 × with 1 × TBS, lysed with 4% (w/v) sodium deoxycholate (SDC) in 100 mM Triethylamonium bicarbonate (TEAB) (pH 8–8.5) and then immediately heated for 5 min at 95 °C to facilitate lysis and inactivate endogenous proteases and phosphatases. Lysates were then sonicated and centrifuged at 16,000 × g for 10 min at 4 °C to clear cell debris, and the protein concentration was determined using BCA reagent (Pierce™, Thermo Fisher Scientific). One hundred μg of each sample was reduced with 5 mM Tris (2-carboxyethyl)phosphine (TCEP) at 55 °C for 30 min, followed by alkylation with 20 mM Chloroacetamide in the dark for 30 min, and digestion with Lys-C and trypsin (1:100) O/N at 37 °C with shaking (1500 rpm). Tryptic digests were acidified with 20% (v/v) trifluoroacetic acid (TFA) to pH 2–3 and centrifuged at 10,000 × g for 10 min at 4 °C to precipitate out the SDC. The supernatant was collected and isobaric tandem mass tag (TMT, Thermo Fisher Scientific) labelled before being combined and desalted with a C18 column (Thermo Fisher Scientific) and eluted with 0.1% (v/v) TFA/40% (v/v) acetonitrile (ACN). Peptides were dried with a SpeedVac and re-suspended in 2% (v/v) ACN/0.1% (v/v) TFA prior to mass spectrometry analysis.

#### TMT labelling

TMT label reagents were used at room temperature, and 41 µl of 100% ACN was added to each isobaric reagent vial to dissolve the TMT label reagent. The different isobaric TMT label reagents were added to each sample and incubate for 1 h at room temperature. The reaction was quenched by adding 8 µl of 5% (v/v) hydroxylamine. Samples were combined at equal amounts prior to desalting on a C18 column for whole proteomic analysis or proceed to phosphopeptide enrichment.

#### Phosphopeptide enrichment

Following TMT labelling and combining all samples, phosphopeptides were enriched as previously described [17]. Briefly, peptides were enriched with a 12:1 TiO2 bead (5010–21,315, GL Sciences, Tokyo, Japan) to protein ratio for 5 min at 40 °C with shaking (2000 rpm). Phosphopeptides were eluted with EP elution buffer (5% (v/v) NH_4_OH in 40% ACN) prior to desalting with in-house prepared SDB-RPS (Empore™, CDS Analyticl, Oxford, PA, USA) stage tip and eluted with 20 μl of 25% (v/v) NH_4_OH in 60% (v/v) ACN and evaporated to dryness in a SpeedVac. The dried peptides were reconstituted in 2% (v/v) ACN/0.3% (v/v) TFA.

#### Mass spectrometry analysis

Samples were analysed on an UltiMate 3000 RSLC nano LC system (Thermo Fisher Scientific) coupled to an Orbitrap Fusion Tribrid mass spectrometer (Thermo Fisher Scientific). Peptides were loaded via an Acclaim PepMap 100 trap column (100 μm × 2 cm, nanoViper, C18, 5 μm, 100 Å, Thermo Fisher Scientific) and subsequent peptide separation was on an Acclaim PepMap RSLC analytical column (75 μm × 50 cm, nanoViper, C18, 2 μm, 100 Å, Thermo Fisher Scientific). One µg of peptide as measured by a nanodrop 1000 spectrophotometer (Thermo Fisher Scientific) was loaded on the pre-column with microliter pickup. Peptides were eluted using a 2 h linear gradient of 80% (v/v) ACN/0.1% FA flowing at 250 nl/min using a mobile phase gradient of 2.5–42.5% (v/v) ACN. The eluting peptides were interrogated using a synchronous precursor selection tandem MS/MS/MS (SPS-MS3) workflow, to eliminate isolation interference and dynamic range compression that are commonly observed in isobaric tag-based quantitative proteomics experiments [18]. For peptide identification, the false discovery rate (FDR) was set to 1% at peptide level. The raw data files were analysed with the MaxQuant Version 1.6.0.16 analysis software using default settings. Group-specific parameters was set to Type Reporter ion MS3 and TMT labels loaded under isobaric labels. Enzyme specificity was set to Trypsin/P and LysC/P, minimal peptide length of 6, and up to 4 missed cleavages were allowed. Search criteria included carbamidomethylation of cysteine as a fixed modification; oxidation of methionine; acetyl (protein N terminus); deamidation (NQ); custom “heavy” deutarated lysine-d8 modification (+ 8.05021396); and phosphorylation of serine, threonine, and tyrosine as variable modifications. The mass tolerance for the precursor was 4.5 ppm and for the fragment ions was 20 ppm. The DDA files were searched against the human UniProt fasta database (v2015–08, 20,210 entries concatenated with DDC^M.tub−KDEL^ and Lyr^M37−KDEL^ peptides). Only lysine containing peptides were included in the CTAP quantitative analysis.

#### Mass spectrometry statistical analysis

Peptide intensities were Log_2_ transformed, imputation via normal distribution with Perseus software before quantile normalisation. The comparison between co-culture/mono-culture was assessed by corresponding fold change (FC) and a two-tailed *t*-test with a *p* < 0.05, and the p values were adjusted for multiple testing using the Benjamini–Hochberg method [19].

#### Functional annotation analysis

Functional annotation and pathway analysis of the (phospho)proteome was conducted using Metascape [20] and Enrichr [21–23]. Overrepresented functional categories among proteins enriched in either mono- or co-cultures (FC > 1.2 for PC3 proteins/phosphosites with raw *p* < 0.05; FC > 2.5 or > 5.0 for WPMY-1 proteins/phosphosites with adjusted *p* < 0.05) were evaluated using hypergeometric distribution with Benjamini Hochberg corrected p value (*p* < 0.01). Criteria for reported functional enrichment required an adjusted p value < 0.01, FDR < 0.05 and > 2 proteins mapping to a functional pathway. Experimentally verified and published protein–protein interactions from STRING [24] and Cytoscape [25] were assessed.

### Analysis of publicly available datasets

Prostate cancer patient mRNA data were obtained through the Multi-Gene Cancer Survival Analysis Tool (http://can-sat.erc.monash.edu) and The Cancer Genomic Atlas (TCGA).

### Statistical analysis

GraphPad Prism 9.0 (GraphPad Software, San Diego, CA, USA) was used for statistical calculations. For all comparisons between two groups, *t*-tests were performed and p value and standard deviation of the mean (SD) were reported. For all comparisons among more than two groups, one-way ANOVA was performed and p values and SD were reported. Results from all in vitro assays are representatives of at least three independent biological repeats unless otherwise specified.

## Results

### The impact of prostate fibroblast co-culture on prostate cancer cell biology

**Fig. 1:**
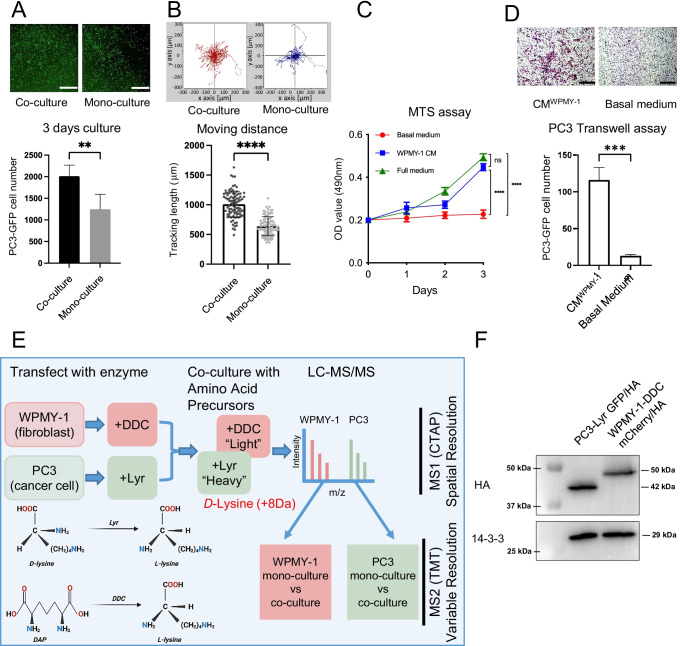
Effect of WPMY-1 prostate fibroblasts on PC3 cell proliferation and migration and application of CTAP to this model system.**A** and **B**, WPMY-1 co-culture increases the proliferation and migration of PC3 cells. Upper panels (**A**) – images of GFP-labelled PC3 cells after mono- or co-culture. The graph provides image quantification. Results of random cell migration assays for PC3 mono-culture and WPMY-1 co-culture (**B**). **C** and **D**, WPMY-1 conditioned medium increases the proliferation and migration of PC3 cells. Results of MTS assay for PC3 cells with either basal medium, full medium or WPMY-1-conditioned medium (**C)** and Transwell assays for PC3 cells with either basal medium or WPMY-1-conditioned medium (**D**). Data are presented as mean ± SD of 3 biological replicates; student *t*-test, **p < 0.01, ***p < 0.001, ****p < 0.0001. Scale bar: 1 mm. **E**, Application of CTAP to the PC3/WPMY-1 model. CTAP provides spatial resolution, identifying which cell type the MS-detected peptides are derived from. Discrimination between additional variables is achieved by differential isobaric tandem mass tag (TMT) labelling of peptides. **F**, Generation of PC3 and WPMY-1 cells expressing HA-tagged Lyr and DDC, respectively. Cell lysates were Western blotted as indicated. CM, conditioned medium

In order to establish a model system to characterising the interaction between prostate cancer fibroblasts and prostate cancer cells, we utilised WPMY-1 immortalised prostate stromal fibroblasts [13], which exhibit a myofibroblast-like phenotype, and PC3 prostate cancer cells, and determined the effects of monolayer co-culture. Co-culture of WPMY-1 cells with PC3 cells led to enhanced proliferation of the latter cells compared to PC3 mono-culture (Fig. 1A), as well as increased random cell motility (Fig. 1B). In addition, conditioned medium from the WPMY-1 cells increased the proliferation and migration of PC3 cells, compared to basal medium (Fig. 1C and D). These data indicated that WPMY-1 co-culture significantly influences the biology of PC cells, but the underpinning mechanisms, as well as the effects of co-culture on the fibroblasts, remained unclear. Therefore, intercellular communication between the cancer cells and fibroblasts in the co-culture system was addressed by CTAP.

### The impact of PC3 co-culture on the WPMY-1 proteome

CTAP allows the proteomes of two different cell types in co-culture to be readily distinguished by a mass spectrometry (MS) workflow [11, 12]. This proteomic strategy provided a solid foundation for resolving intercellular signalling pathways within the prostate TME (Fig. 1E). Briefly, PC3 and WPMY-1 cells were transduced with optimised lysine racemase (Lyr) GFP and optimised diaminopimelate decarboxylase (DDC) mCherry respectively, selected with SILAC + D-lysine or SILAC + DAP medium and then subjected to fluorescence-activated cell sorting (Supplementary Fig. 1A). After selecting, the PC3-Lyr GFP cells stably expressed Lyr^M37− KDEL^, enabling utilisation of D-lysine as a L-lysine source, while the WPMY-1-DDC mCherry cells expressed DDC^M.tub−KDEL^, enabling utilisation of DAP as a L-lysine source (Fig. 1D). Expression of the appropriate enzymes was confirmed by Western blot analysis (Fig. 1F). CTAP was then combined with isobaric TMT labelling and MS-based (phospho)proteomic workflows to elucidate signalling between PC3 and WPMY-1 cells in a co-culture system compared to corresponding mono-cultures (Fig. 1D, Supplementary Fig. 1B).

**Fig. 2:**
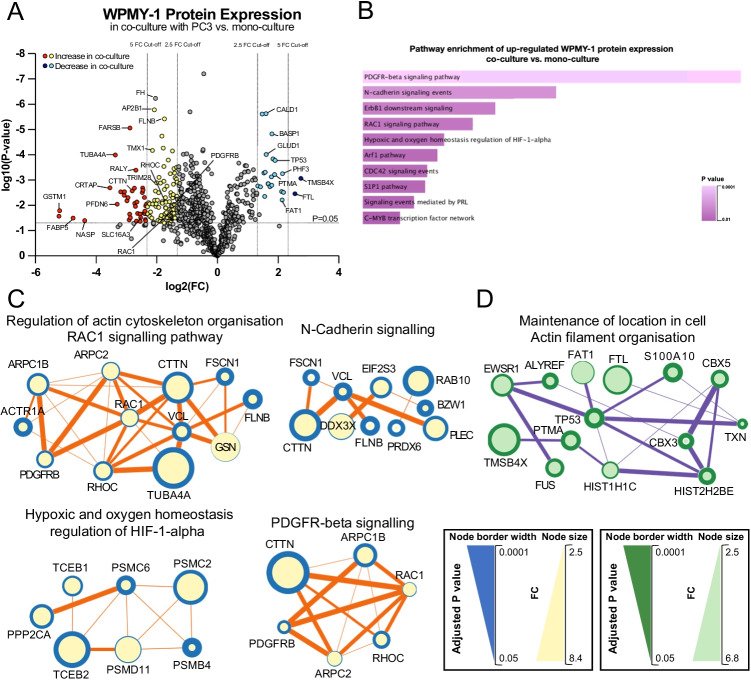
Profiling of the WPMY-1 proteome in PC3 co-culture versus mono-culture.** A**, Volcano plot of differentially enriched WPMY-1 proteins in co-culture versus mono-culture. Proteins that increased in abundance upon co-culture with PC3 by a FC > 2.5 are represented by yellow dots and those with FC > 5 are represented by red dots. Proteins that decreased in abundance upon co-culture with PC3 by FC > 2.5 and FC > 5 are represented by light and deep blue dots, respectively. **B**, Cell signalling pathway enrichment of up-regulated WPMY-1 proteins in co-culture. Data from NCI-nature pathway database. **C** and **D**, Major protein interaction networks formed by proteins with increased (**C**) and decreased (**D**) abundance in co-culture. Analysis was undertaken using STRING/Cytoscape. The node size indicates the fold change (FC) for co-culture versus mono-culture and the node border width indicates the inverse adjusted p value. Proteins with higher FC and lower adjusted p value are represented by larger nodes with wider border. Node connection line thickness is representative of STRING score between connected proteins

In the global proteomic analysis of WPMY-1 cells, using a 2.5 fold change (FC) cut-off, 125 WPMY-1 proteins were significantly up-regulated in PC3 co-culture and 25 proteins were down-regulated, reduced to 25 proteins significantly up-regulated and two proteins down-regulated with a five fold cut-off (Fig. 2A, Supplementary Table S1). Functional pathway analysis revealed WPMY-1 proteins with significantly increased abundance in co-culture were involved with signalling by PDGFR-beta, N-cadherin, ErbB1/EGFR, HIF-1alpha, RAC1 and CDC42 (Fig. 2B, Supplementary Table S2). Proteins with significantly decreased abundance in co-culture were involved in categories that included ‘regulation of DNA metabolic process’, ‘chromatin assembly’, ‘maintenance of location in the cell’ and ‘actin filament organisation’ (Supplementary Fig. 2A, Supplementary Table S2). Both up- and down-regulated proteins were enriched for ‘VEGFA-VEGFR2 signalling pathway’ (Supplementary Fig. 2A, Supplementary Table S2).

STRING/Cytoscape network analysis of proteins with increased expression in co-culture identified hubs involved in ‘regulation of actin cytoskeleton organisation’ and ‘RAC1 signalling pathway’ (e.g. RAC1, RHOC, CTTN, PDGFRB, ARPC1B and ARPC2), ‘N-Cadherin signalling’ (e.g. CTTN, RAB10, FSCN1 and PLEC), ‘hypoxic and oxygen homeostasis regulation of HIF-1-alpha’ (e.g. TCEB1, TCEB2, PPP2CA and PSMC6) and ‘PDGFR-beta signalling’ (e.g. CTTN, PDGFRB, RHOC and RAC1) (Fig. 2C, Supplementary Table S2). The increased expression of PDGFRB in co-cultured WPMY-1 cells (2.64 FC over mono-culture, p = 0.0095) (Supplementary Table S1) is of note because PDGFRB is commonly regarded as a CAF marker [26]. Network analysis of proteins with significantly decreased expression in WPMY-1 cells revealed a prominent interaction hub involved in ‘maintenance of location in cell’ and ‘actin filament organisation’, functional categories linked to cell polarisation, cell migration and invasion. These proteins included FTL, TMSB4X, TXN, TP53, ALYREF, S100A10 and PTMA (Fig. 2D, Supplementary Table S2). Interestingly, the actin binding protein TMSB4X was the most markedly down-regulated fibroblast protein in co-culture (6.72 FC compared to mono-culture, p = 0.0009).

**Fig. 3:**
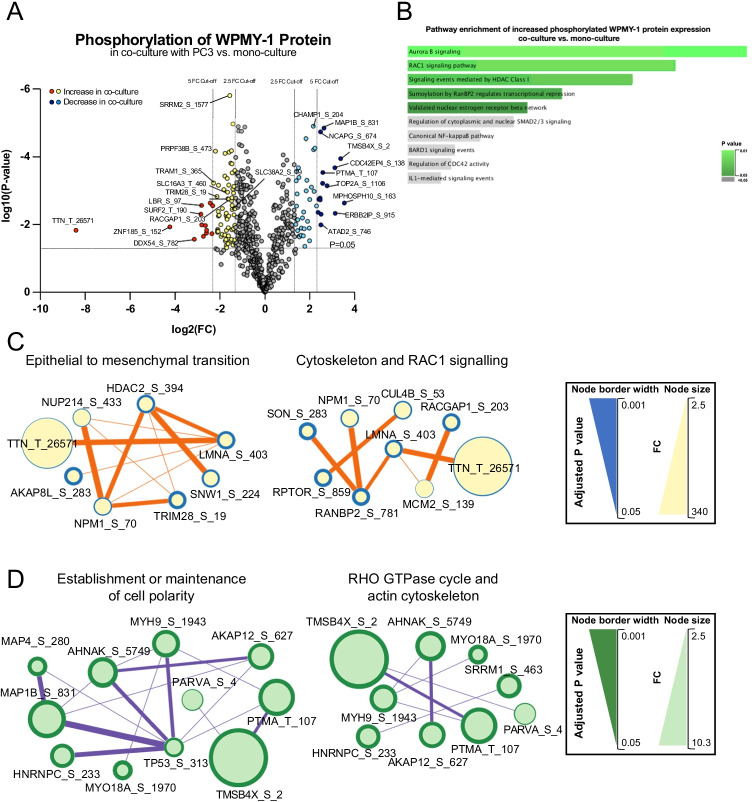
Differentially abundant phosphosites of WPMY-1 proteins in co-culture with PC3 versus in mono-culture.** A**, Volcano plot showing differentially abundant phosphosites of ‘light’ WPMY-1 proteins in co-culture versus mono-culture. Increased phosphosites with a FC > 2.5 are represented by yellow dots; and FC > 5 phosphosites are represented by red dots. Phosphosites that decreased in abundance upon co-culture with PC3 by FC > 2.5 and FC > 5 are represented by light and deep blue dots, respectively. **B**, Cell signalling pathway enrichment of up-regulated WPMY-1 phosphoproteome in co-culture. Data from NCI-nature pathway database. **C** and **D**, Networks of proteins with increased (**C**) and decreased (**D**) phosphorylation abundance in WPMY-1 cells. Analysis was undertaken using STRING/Cytoscape

In addition, a TiO2-enrichment workflow was utilised to identify phosphorylation sites in WPMY-1 cells affected by co-culture. Using a 2.5 FC cut-off value, 75 proteins displayed significantly increased phosphorylation upon co-culture with PC3, while 47 proteins showed decreased phosphorylation, while using a 5 FC cut-off value, 13 phosphosites were significantly up-regulated and 15 phosphosites were down-regulated (Fig. 3A, Supplementary Table S3).

Functional analysis of WPMY-1 proteins exhibiting increased phosphosite abundance in co-culture revealed enrichment for categories associated with ‘Aurora B signalling’, ‘RAC1 signalling pathway’ ‘RHOC GTPase cycle’, ‘mitotic cell cycle’ and ‘epithelial to mesenchymal transition (EMT)’. Analysis of sites with decreased abundance in co-culture revealed enrichment for categories associated with ‘establishment or maintenance of cell polarity’, ‘cellular response to cytokine stimulus’ and ‘RHO GTPase cycle’ (Fig. 3B, Supplementary Fig. 2B, Supplementary Table S2).

Network analysis using STRING/Cytoscape for WPMY-1 proteins with increased phosphorylation in co-culture revealed prominent interaction hubs involving ‘epithelial to mesenchymal transition’, ‘cytoskeleton’ and ‘RAC1 signalling’, and for phosphosites with decreased abundance, interaction hubs associated with ‘establishment or maintenance of cell polarity’, ‘RHO GTPase cycle’ and ‘actin cytoskeleton’ (Fig. 3C-D, Supplementary Table S2). When integrating functional pathway enrichments for modulation of the WPMY-1 proteome and phosphoproteome upon co-culture, there was a notable enrichment for regulation of EMT, cell polarity, PDGFRB and Rho GTPase signalling and the actin cytoskeleton.

### The impact of WPMY-1 co-culture on the PC3 proteome

While we identified significant impacts of PC3 cancer cells on the (phospho)proteome of WPMY-1 fibroblasts, intercellular communication between cancer cells and CAFs is known to be bi-directional [27, 28]. Consequently, we exploited the ability of CTAP to discriminate both cell proteomes in co-culture and interrogated the effect of WPMY-1 co-culture on the PC3 cells.

**Fig. 4:**
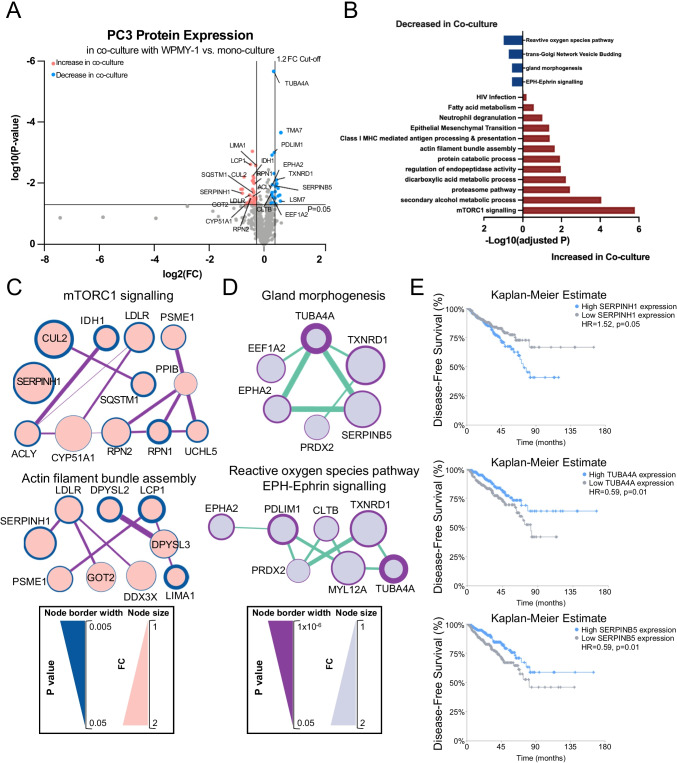
Profiling of the PC3 proteome in WPMY-1 co-culture versus mono-culture.** A**, Volcano plot highlighting differentially expressed PC3 proteins across the two culture conditions. Proteins that increased in abundance upon co-culture with WPMY-1 are represented by pink dots. Proteins that decreased in abundance upon co-culture with WPMY-1 are represented by blue dots. **B**, Functional categories associated with differentially abundant PC3 proteins in co-culture compared to mono-culture. **C** and **D**, Major protein interaction networks formed by proteins with increased (**C**) and decreased (**D**) abundance in co-culture. Analysis was undertaken using STRING/Cytoscape. **E**, Relationship of SERPINH1, TUBA4A and SERPINB5 with disease-free survival of PC patients using data derived from the TCGA (50% cut-off expression level). Figures are adopted from the Multi-Gene Cancer Survival Analysis Tool (http://can-sat.erc.monash.edu)

In general, modulation of the PC3 (phospho)proteome was more modest than that observed for the WPMY-1 cells, and so a more relaxed 1.2 FC was used as the cut-off (see Methods). Applying this approach, 36 proteins were significantly up-regulated in co-culture and 25 proteins down-regulated (Fig. 4A, Supplementary Table S4). Functional analysis revealed that PC3 proteins increased in co-culture were enriched for pathways including ‘mTORC1 signalling’, ‘actin filament bundle assembly’ and ‘epithelial mesenchymal transition’, consistent with fibroblast co-culture increasing the proliferation and migration of PC3 cells (Fig. 1A-D), as well as protein catabolism and antigen processing/presentation (Fig. 4B, Supplementary Table S5). Functional pathways for PC3 proteins that decreased in co-culture were associated with ‘Reactive oxygen species pathway’, ‘gland morphogenesis’ and ‘EPH-Ephrin signalling’ (Fig. 4B, Supplementary Table S5). The latter represents a key regulator of cancer cell adhesion, motility and cell matrix interactions [29].

STRING/Cytoscape network analysis of proteins with increased expression in co-culture identified hubs involved in ‘mTORC1 signalling’ (e.g. SERPINH1, SQSTM1, RPN1 and LDLR) and ‘Actin filament bundle assembly’ (e.g. SERPINH1, DPYSL2, DPYSL3, LCP1 and LIMA1) (Fig. 4C, Supplementary Table S5). Network analysis of proteins with significantly decreased expression in PC3 cells revealed a prominent interaction hub involved in ‘gland morphogenesis’ (e.g. TUBA4A, SERPINB5, TXNRD1) and interconnected hubs representing ‘Reactive oxygen species pathway’ and ‘EPH-Ephrin signalling’ (e.g. EPHA2, TUBA4A, PDLIM1 and TXNRD1) (Fig. 4D, Supplementary Table S5).

Of note, SERPINH1, involved in both up-regulated hubs (Fig. 4C), exhibits an oncogenic role in breast cancer, where it acts to promote deposition of specific collagens and fibronectin [30]. Since SERPINH1 exhibits higher expression in PCs compared to benign tissue [31], we interrogated how its expression is related to patient prognosis using publicly-available TCGA data. Importantly, high SERPINH1 expression positively correlated with reduced disease-free survival (Fig. 4E). In contrast, TUBA4A, a component of the microtubule cytoskeleton [32] was involved in both of the down-regulated networks (Fig. 4D), and reduced expression of TUBA4A positively associated with poor prognosis in PC patients, suggesting a potential tumour suppressor role (Fig. 4E). In addition, low expression of SERPINB5, a component of the down-regulated ‘gland morphogenesis’ network and a protein that exhibits context-dependent oncogenic and tumour suppressor roles [33] also positively correlated with reduced disease-free survival in PC (Fig. 4E).

**Fig. 5:**
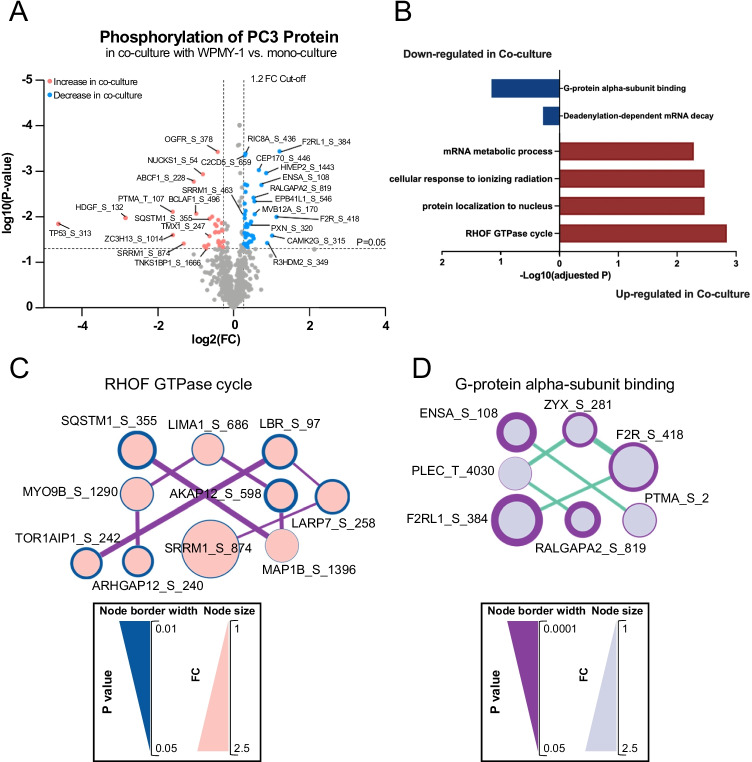
Profiling of the PC3 phosphoproteome in WPMY-1 co-culture versus mono-culture.** A**, Volcano plot for differentially phosphorylated PC3 proteins across the two culture conditions. The differentially phosphorylated proteins that increased in abundance upon co-culture with WPMY-1 are represented by pink dots, and the differentially phosphorylated proteins that decreased in abundance upon co-culture with WPMY-1 represented by blue dots. **B**, Functional categories associated with differentially phosphorylated PC3 proteins in co-culture compared to mono-culture. **C** and **D**, Networks of proteins with increased (**C**) and decreased (**D**) phosphorylation abundance in PC3 cells. Analysis was undertaken using STRING/Cytoscape

The CTAP approach also enabled characterisation of the PC3 phosphoproteome in co-culture, with 30 sites significantly up-regulated and 40 sites down-regulated (Fig. 5A, Supplementary Table S6). Functional analysis of proteins exhibiting increased and decreased phosphosite abundance revealed enrichment for the category ‘RHOF GTPase cycle’ and ‘G-protein alpha-subunit binding’, respectively (Fig. 5B, Supplementary Table S5). Interaction hubs for these categories are presented in Fig. 5C-D. The effect on RHOF signalling is consistent with the enhanced migration of the PC3 cells in co-culture, since RHOF regulates actin cytoskeleton organisation, including filopodia formation [34]. In addition, down-regulation of RALGAPA2, a component of the ‘G-protein alpha-subunit binding’ network, enhances migration and invasion of PC cells and is associated with progression of prostatic intraepithelial neoplasia to PC [35].

### TMSB4X regulates PC3/CAF intercellular communication and CAF function in the TME

**Fig. 6:**
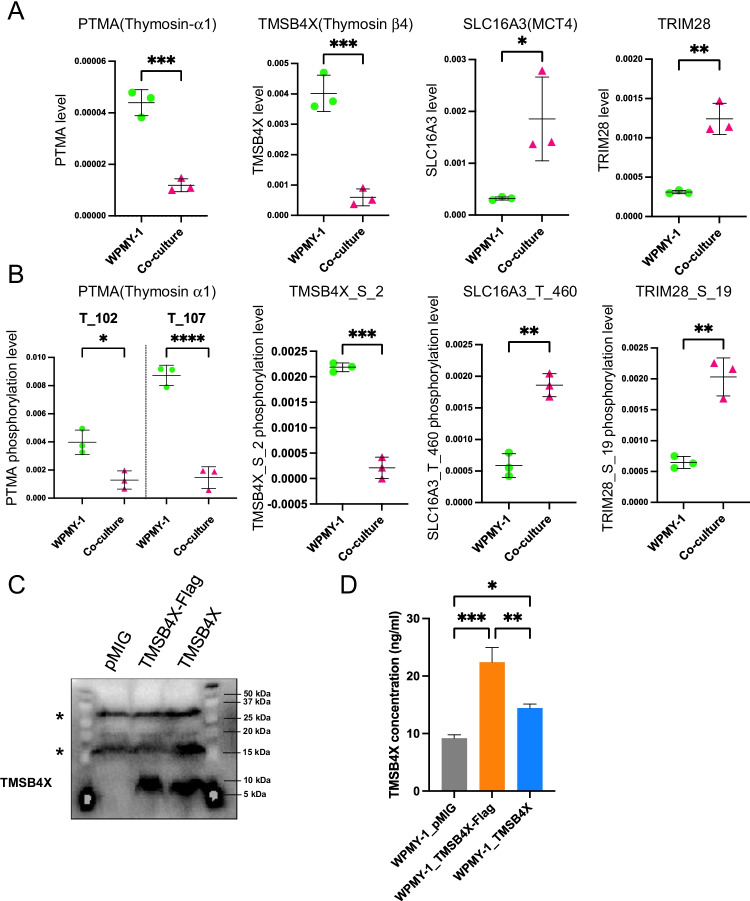
Proteinswith differential total expression and phosphosite abundance in co-cultures versus mono-cultures. Quantification analysis of PTMA, TMSB4X, SLC16A3 and TRIM28 in the proteomic (**A**) and phosphoproteomic (**B**) datasets. Each data point represents a biological replicate. Data are presented as mean ± SD; Two-tailed *t*-test; *p < 0.05, **p < 0.01, ***p < 0.001, ****p < 0.0001. **C**, Ectopic expression of TMSB4X in HEK293 cells. Cells were transduced with different constructs and cell lysates subjected to Western blotting as indicated. *, non-specific bands. **D**, Overexpression of TMSB4X in WPMY-1 cells. TMSB4X was assayed in cell lysates using a specific TMSB4X ELISA

Close inspection of the CTAP datasets revealed that some proteins in WPMY-1 cells exhibited altered expression at both the total protein (Fig. 6A) and phosphosite level (Fig. 6B). These included PTMA (Thymosin α1), TMSB4X (Thymosin β4), SLC16A3 (MCT4) and TRIM28. Among them, the protein levels of PTMA and TMSB4X were decreased in co-culture compared to mono-culture, and decreased phosphorylation at PTMA T102, PTMA T107 and TMSB4X S2 was also detected. Of note, while the decrease in PTMA T102 and TMSB4X S2 phosphorylation was similar in magnitude to the change in protein levels, the decrease in phosphorylation of PTMA T107 was greater (approximately nine fold compared to four fold), indicating a change in relative phosphorylation on this site. In contrast, increased expression of SLC16A3 and TRIM28 was identified in co-culture with comparable increases in SLC16A3 T460 and TRIM28 S19 phosphorylation. Since TMSB4X was the only protein to decrease > five fold in both the whole proteome and phosphorylated proteome datasets under co-culture conditions and is a member of the actin-binding protein family with known roles in regulating organisation of the actin cytoskeleton [36, 37], we selected this protein for further analysis.

### Overexpression of TMSB4X in WPMY-1 cells decreases migration of both PC3 and WPMY-1 cells in co-culture

In order to characterise the biological effect of TMSB4X, WPMY-1 cells overexpressing TMSB4X were generated to oppose the down-regulation of WPMY-1 TMSB4X that occurs upon CAF co-culture. Western blotting demonstrated that HEK293T transfected with the TMSB4X construct expressed a higher level of TMSB4X compared to the vector control (Fig. 6C). In addition, introduction of the construct into WPMY-1 cells and then assay of cell lysates using a TMSB4X ELISA confirmed expression in the transduced cells (Fig. 6D) and indicated that the WPMY-1_TMSB4X-Flag cell line exhibited the highest level of TMSB4X. Consequently, this cell line was used for functional experiments.

**Fig. 7:**
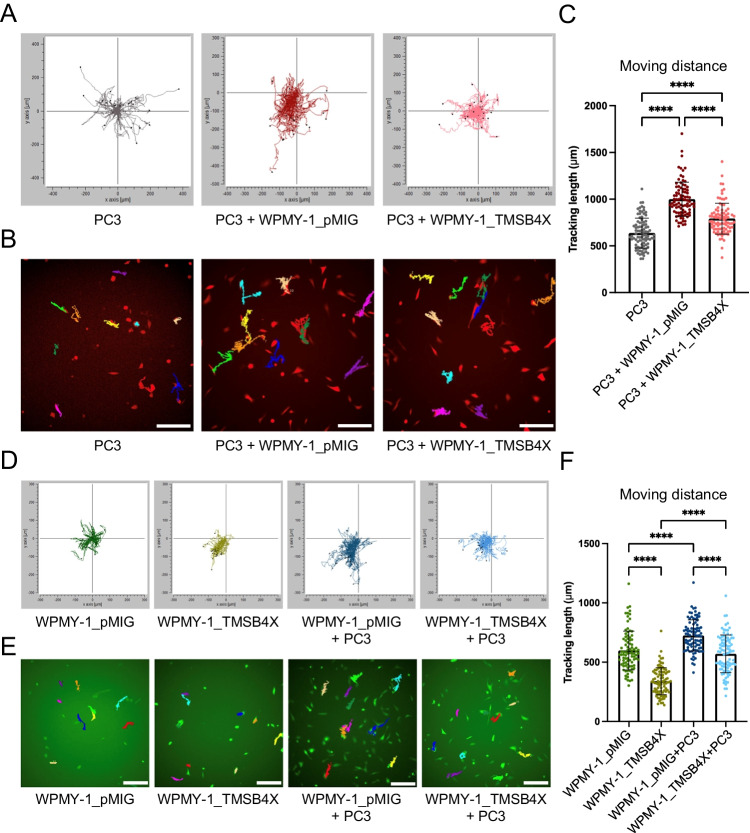
Effect of TMSB4X overexpression in WPMY-1 cells on cell motility.** A**, Effect on PC3 cells. Trajectories of PC3 cells alone or co-cultured with WPMY-1_pMIG and WPMY-1_TMSB4X. **B**, Examples of dragon tails display showing single PC3 cell migration tracks in which temporal changes in cell location are indicated by coloured lines (last frames of the time-lapse movies). **C**, Quantification of the accumulative migrated distance which PC3 cells travelled when cultured alone as well as in the different co-culture systems. **D** Effect on WPMY-1 cells. Trajectories of WPMY-1 cells, ± TMSB4X, in mono-culture and co-cultured with PC3 cells. **E**, Examples of dragon tails display showing single WPMY-1 cell migration tracks. **F**, Quantification of the accumulative migrated distance which WPMY-1 cells travelled when cultured alone as well as in the co-culture system. Each data point represents a single cell that has been analysed in the time-lapse movies. Data are presented as mean ± SD; one-way ANOVA, Tukey’s multiple comparisons; ****p < 0.0001. Scale bar: 100 μm

In order to interrogate the potential function of TMSB4X in the prostate TME, a random cell migration assay was employed. Briefly, PC3 cells labelled with mCherry fluorescence and control or TMSB4X-overexpressing WPMY-1 cells labelled with GFP were cultured either alone or together. Cells were allowed to randomly migrate for 24 h and accumulative distance (the total distance that one single cell travels within a certain time) recorded and analysed by time-lapse microscopy. Co-culture of PC3 with either WPMY-1_pMIG or WPMY-1_TMSB4X cells significantly increased the accumulative migrated distance of PC3 cancer cells compared to PC3 culture alone (Fig. 7A-C). However, overexpression of TMSB4X in WPMY-1 cells significantly attenuated the pro-migratory effect of fibroblast co-culture on the PC3 cells (Fig. 7C).

On the other hand, overexpression of TMSB4X resulted in reduced WPMY-1 cell motility (Fig. 7D-F). Furthermore, while co-culture with PC3 cells significantly increased the accumulative migrated distance of WPMY-1 cells, this effect was reduced for the WPMY-1_TMSB4X cells, although the motility of the latter cells in co-culture was still significantly higher than in mono-culture.

### Overexpression of TMSB4X protects WPMY-1 cells from expressing CAF markers upon co-culture with PC3 cells

**Fig. 8:**
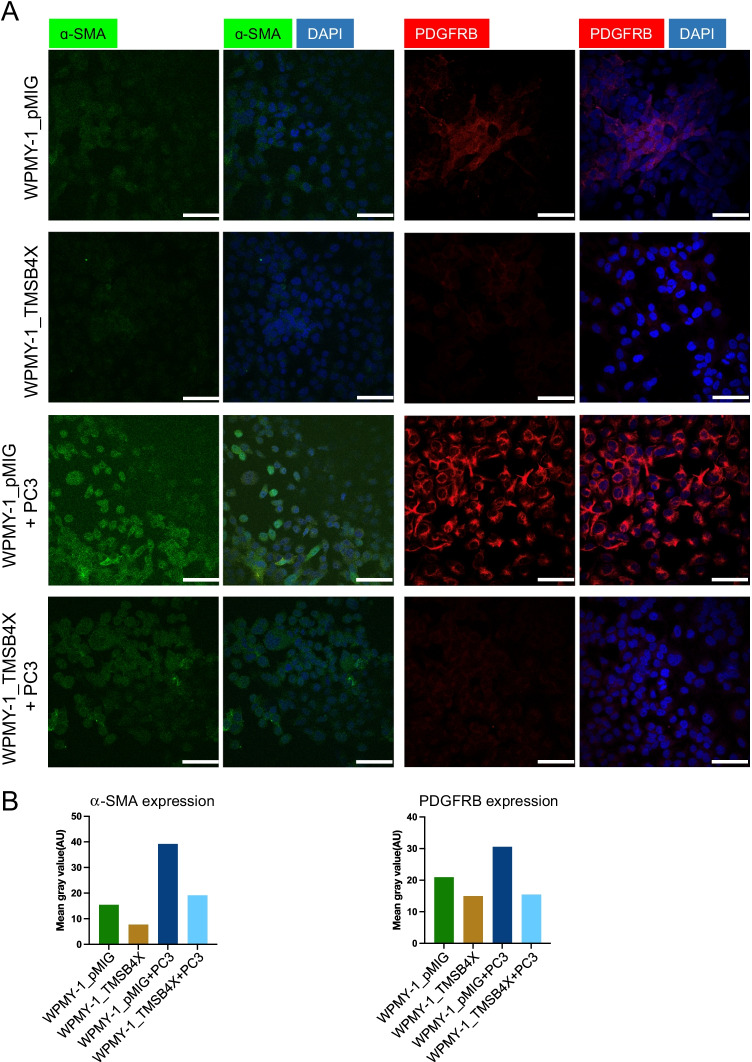
Effect of TMSB4X on expression of CAF markers by WPMY-1 cells.** A**, Effect on α-SMA and PDGFRB expression. Representative immunofluorescence images of WPMY-1_pMIG and WPMY-1_TMSB4X cells in mono-culture and in co-culture with PC3 cells. Nuclei were stained with DAPI in blue, and α-SMA and PDGFRB were stained green and red, respectively. **B**, Quantification of α-SMA and PDGFRB expression. Y axis represents the mean fluorescence intensity. Scale bar: 30 μm

Immunofluorescence staining was used to characterise the expression of CAF markers in the WPMY-1 cells (Fig. 8, Supplementary Fig. 3–4). Expression of both α-SMA and PDGFRB was reduced in WPMY-1-TMSB4X cells compared to controls (Fig. 8A-B, Supplementary Fig. 3A-B and Supplementary Fig. 4). In addition, while co-culture with PC3 cells increased α-SMA and PDGFRB expression in WPMY-1 cells, this increase was largely blocked in the TMSB4X-overexpressing cells. These data support a model whereby overexpression of TMSB4X protects prostate fibroblasts from being re-programmed to a CAF-like phenotype by PC cells.

## Discussion

This study represents the first time that CTAP has been applied to interrogate reciprocal signalling between PC cells and prostate fibroblasts, a process known to promote PC progression [3–5]. By defining changes in cell type-specific (phospho)proteomes upon co-culture, the approach revealed mechanisms that underpin particular phenotypic changes and biological responses, and also identified candidate prognostic biomarkers. In addition, this study identified the actin-binding protein TMSB4X as a key regulator of not only the fibroblast phenotype, acting to suppress transition to a CAF-like phenotype in co-culture, but also a facilitator of intercellular communication between the fibroblasts and PC cells.

Analysis of signalling network changes in the WPMY-1 and PC3 cells upon co-culture revealed similarities and differences. Consistent with the enhanced migration of both cell types, major altered functional categories included the actin cytoskeleton and signalling by Rho family GTPases, but the detailed mechanisms differed. For example, up-regulation of actin-related protein (ARP) 2/3 complex subunits 1B and 2 (ARPC1B, ARPC2) was detected in WPMY-1 but not PC3 cells, while changes to Rho family signalling included increased expression of RAC1 and RHOC in the former cells, and enhanced phosphorylation of RHOF pathway components in the latter. In terms of tyrosine kinase signalling, PDGFRB and its associated pathway was up-regulated in the WPMY-1 cells, while EPHA2 and EPH-Ephrin signalling was down-regulated in the PC3s. Furthermore, while proliferative signalling was impacted in both cell types, this reflected changes in AURKB and mitotic cell cycle in the fibroblasts, and mTORC1 signalling in the PC cells. These findings likely reflect the contrasting biology and signalling architecture of the co-cultured cell types and also indicate that therapeutic targeting of the different cell types may require different approaches. In addition, application of CTAP revealed candidate cell type-selective prognostic biomarkers. In the case of the fibroblasts, increased expression of PDGFRB occurred upon co-culture, and PDGFRB status of the prostate stroma has previously been identified as an independent prognostic factor for clinical and biochemical recurrence in PC [38]. However, our work also identified SERPINH1, TUBA4A and SERPINB5 as proteins exhibiting associations with patient prognosis consistent with their expression changes in co-cultured PC3 cells, highlighting these as prognostic biomarker candidates for validation in independent patient cohorts.

A further important finding was the identification of fibroblast TMSB4X has a key regulator of communication between these cells and co-cultured PC cells. TMSB4X regulates diverse biological endpoints across different cell types including wound healing, fibrosis, inflammation and cellular differentiation [36]. In terms of mechanism, one of the best-characterised properties of TMSB4X is its ability to bind and sequester G actin monomers, and thereby influence actin filament dynamics and localisation [37]. In terms of the role of TMSB4X in cancer, most studies have focused on its role in cancer cells, where in general, TMSB4X plays a pro-migratory role, drives EMT and positively associates with poor prognosis [39–42]. However, in hepatic stellate cells, which are mesenchymal, TMSB4X acts to suppress cell migration and stellate cell activation, as determined by $\alpha$-SMA expression [43, 44]. The latter results are consistent with our findings for WPMY-1 cells. Possible mechanisms that may underpin the inhibitory effect of TMSB4X on cell migration include its ability to modulate actin network dynamics [37] and inhibit TGF$\upbeta$ and PDGF signalling by altering microtubule organisation and promoting PDGFRB down-regulation, respectively [42, 45, 46]. In this context, the possibility that TMSB4X might negatively regulate PDGF signalling by promoting endocytic down-regulation of PDGFRB [46] is supported by the enhanced PDGFRB expression in co-cultured WPMY-1 cells, which have markedly reduced TMSB4X levels, and reversal of this effect by TMSB4X overexpression. Furthermore, a phosphorylation of TMSB4X S2 site was identified in the phosphoproteomic analysis, which was also found in studies of other cells and cancers [47]. Although the function of decreased phosphorylation of TMSB4 S2 is still unclear, it is possibly associated with the steric and allosteric components of TMSB4X/profilin exchange process that is important for creating an effective way of tuning the rate and extent of actin polymerisation [48].

Our integrated proteomic, functional and cell imaging analyses indicated that co-culture of WPMY-1 and PC cells re-programmes the former cells to a CAF-like phenotype characterised by enhanced PDGFRB and $\alpha$-SMA expression, and that this conversion is opposed by TMSB4X. However, overexpression of TMSB4X in the WPMY-1 cells decreased the migratory potential of not only the WPMY-1 cells but also co-cultured PC3s. Since TMSB4X can be secreted [36], one potential explanation is secreted TMSB4X acting in a paracrine manner on the PC3s. However, since TMSB4X generally has a pro-migratory effect on malignant epithelial cells, a more likely explanation is altered production of other secreted factors by the fibroblasts. One such candidate factor is SERPINE1 (PAI1), which is known to promote cell adhesion, spreading and migration, and is positively regulated by TGF$\upbeta$ signalling [49–51]. SERPINE1 was markedly up-regulated in co-cultured WPMY-1 cells where it may promote migration of both cell types, but this mechanism may be opposed by the known inhibitory effect of TMSB4X on TGF$\upbeta$ action [45].

Overall, our findings highlight the power of utilising CTAP to decipher intercellular communication in the TME. While this study has utilised PC3 cells, which model metastatic aggressive-variant, castrate-resistant PC with a defined set of genetic alterations including TP53 mutation and PTEN loss [51], extension to other stages of disease progression and mutational profiles is likely to yield further insights that can be exploited in the context of precision oncology. In addition, recent developments in our understanding of CAF heterogeneity will inform additional fibroblast subtypes that can be interrogated by the CTAP approach.

## Conclusions

Use of CTAP-MS revealed signalling network changes that occur upon co-culture of PC cells and prostatic fibroblasts, demonstrating changes to mTOR signalling and the actin cytoskeleton in the former cells, and transition to a CAF-like phenotype in the latter. The approach also identified potential prognostic biomarkers in the PC cells, and fibroblast TMSB4X was functionally characterised as a key regulator of intercellular communication that opposes fibroblast reprogramming. Overall, the study highlights the power of CTAP-MS to resolve intercellular communication in cancer and identify novel biomarkers and therapeutic targets.

## Supplementary Information

Below is the link to the electronic supplementary material.

Supplementary file1 (DOCX 12154 KB)

Supplementary file2 (XLSX 1068 KB)

## Data Availability

MS data will be made available from the ProteomeXchange Consortium via the PRIDE partner repository [52]. Other raw data can be obtained from the corresponding author.

## Abbreviations

CAFs: Cancer-associated fibroblasts
TME: Tumour microenvironment
CTAP: Cell type-specific labelling with amino acid precursors
TMSB4X: Thymosin β4
PC: Prostate cancer
ECM: Extracellular matrix
EMT: Epithelial-mesenchymal transition
LOXL2: Lysyl oxidase-like 2
MS: Mass spectrometry
ELISA: Enzyme-linked immunosorbent assay
FDR: False discovery rate
FC: Fold change
TCGA: The Cancer Genomic Atlas
CM: Conditioned medium
DDC: Diaminopimelate decarboxylase
Lyr: Lysine
ARP: Actin-related protein
