# Supplementary material for: Proteomic characterisation of prostate cancer intercellular communication reveals cell type-selective signalling and TMSB4X-dependent fibroblast reprogramming
Source: Cell Oncol (Dordr). 2022 Sep 28;45(6):1311–28. doi: 10.1007/s13402-022-00719-z (PMC9747870; doi:10.1007/s13402-022-00719-z)
Supplement: Supplementary file 1 — Supplementary file1 (DOCX 12154 KB) [file 13402_2022_719_MOESM1_ESM.docx]

**Proteomic characterisation of prostate cancer intercellular communication reveals cell type-selective signalling and TMSB4X-dependent fibroblast reprogramming**

Yunjian Wu, Kimberley C. Clark, Elizabeth V. Nguyen, Birunthi Niranjan, Lisa G. Horvath, Renea A. Taylor and Roger J. Daly.

**Supplementary Figures and Tables**


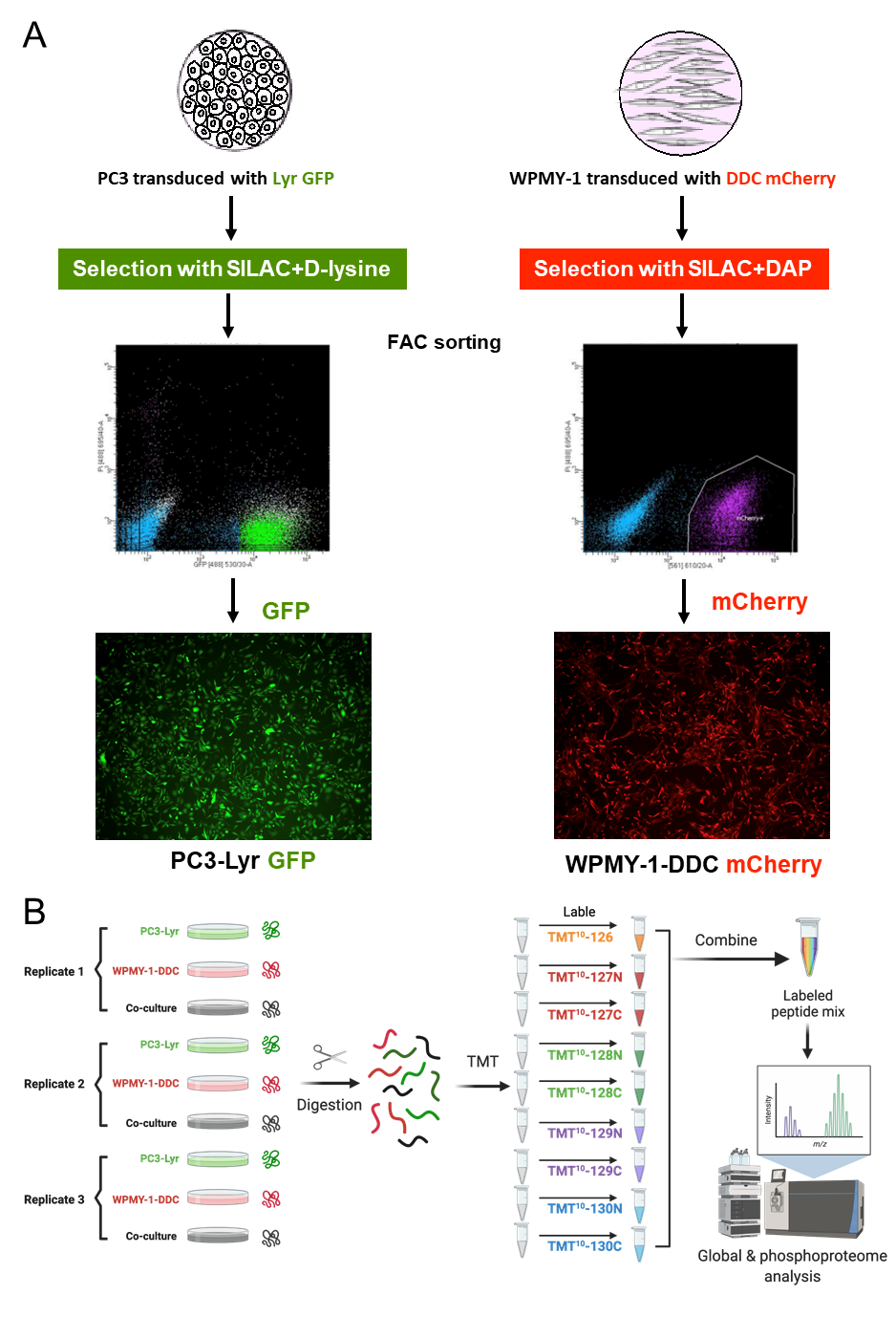


**Supplementary Fig. S1 Application of CTAP to the PC3-WPMY-1 intercellular communication model.** **A**, Workflow for generating the PC3-Lyr GFP cells and WPMY-1-DDC mCherry cells used in CTAP. **B**, TMT labelling strategy and CTAP mass spectrometry (MS) experiment workflow. Lyr, lysine racemase. DDC, diaminopimelate decarboxylase. DAP, meso-2,6-diaminopimelate.

**Supplementary Fig. S2** **Functional analysis of differentially abundant WPMY-1 proteins and phosphosites in co-culture with PC3 cells versus monoculture.** **A**, Global functional annotation of differentially abundant WPMY-1 proteins in co-culture compared to mono-culture. **B**, Global functional annotation of differentially abundant WPMY-1 phosphosites in co-culture compared to mono-culture. Analysis data were extracted from Metascape ^21^.

**Supplementary Fig. S3** **Effect of TMSB4X on expression of CAF markers by WPMY-1 cells (Secondary replication). A**, Effect on α-SMA and PDGFRB expression. Representative immunofluorescence images of WPMY-1_pMIG and WPMY-1_TMSB4X cells in mono-culture and in co-culture with PC3 cells. Nuclei were stained with DAPI in blue, and α-SMA and PDGFRB were stained green and red, respectively. **B**, Quantification of α-SMA and PDGFRB expression. Y axis represents the mean fluorescence intensity. Scale bar: 30 μm.

**Supplementary Fig. S4 CAF markers expression on WPMY-1 cells upon co-culture with PC3 cells.** Effect on α-SMA and PDGFRB expression. Representative immunofluorescence images of PC3 cells in mono-culture and in co-culture with WPMY-1_pMIG cells. Nuclei were stained with DAPI in blue, and α-SMA and PDGFRB were stained green and red, respectively. Scale bar: 30 μm.
